# Physical activity and non-movement behaviours: their independent and combined associations with metabolic syndrome

**DOI:** 10.1186/s12966-016-0350-5

**Published:** 2016-02-19

**Authors:** Deepa P. Rao, Heather Orpana, Daniel Krewski

**Affiliations:** Interdisciplinary School of Health Sciences, University of Ottawa, Ottawa, ON Canada; School of Psychology, University of Ottawa, Ottawa, ON Canada; School of Epidemiology, Public Health and Preventive Medicine, University of Ottawa, Ottawa, ON Canada

**Keywords:** Metabolic syndrome, Sedentary behaviours, Non-movement behaviours, Physical activity guideline adherence, Chronic disease risk

## Abstract

**Background:**

Metabolic syndrome (MetS) is a prevalent risk condition associated with a higher risk of chronic conditions, including diabetes and cardiovascular diseases. Physical activity and non-movement behaviours (NMB), including sleep, screen time and sedentary activity, have been associated with MetS. In light of the increasing prevalence of NMBs, and the moderate rates of physical activity guideline adherence in Canada, this analysis examines the independent and combined associations of NMB and physical activity with MetS.

**Methods:**

Data on Canadians 18 years and older from the Canadian Health Measures Survey (*n* = 2901) were used to examine the moderating effect of moderate-to-vigorous physical activity (MVPA) guideline adherence (150 minutes or more of MVPA/week, based on accelerometer) on the association of NMBs (sleep based on self-report, screen time based on self-report, and sedentary time based on accelerometer) with MetS. Logistic regression analyses were conducted and sampling weights were applied to represent the Canadian adult population.

**Results:**

A graded association between PA and MetS was observed, with those achieving less MVPA than guidelines having a higher odds of MetS (OR 2.9, 95 % CI: 1.9–4.5 for < 75 mins/week of MVPA, and OR 1.8, 95 % CI: 1.2–2.8 for 75–150 mins/week, as compared to those accumulating 150 mins/week or more). When examining the moderating effect of PA on the association between NMBs and MetS, we found that (1) for participants who met guidelines, no level of any NMB was significantly associated with MetS and (2) for those who did not achieve guidelines, there was an increased odds of MetS based on excess NMB time(OR 3.2, 95 % CI: 1.5–6.8 for 1.4–2.1 h/day and OR 4.4, 95 % CI: 2.5–7.9 for ≥2.1 h/day of screen time and 75–150 mins/week of MVPA, OR 1.7, 95 % CI: 1.1–2.5 for ≥8 h/day of sleep time and <75 mins/week of MVPA, and OR 2.2, 95 % CI: 1.3–3.8 for 9.2–10.3 h/day of sedentary time and <75 mins/week of MVPA).

**Conclusions:**

Adhering to physical activity guidelines may mitigate the associations of NMBs with MetS. Given the novel findings that associations between NMBs and MetS were not significant among Canadians meeting PA guidelines, these results suggest the beneficial role of physical activity to prevent chronic disease risk.

## Background

**Fig. 1:**
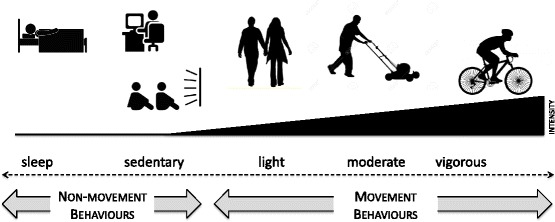
Range of movement and non-movement behaviours over a 24 hour day

Over the past several decades, data have demonstrated a change in the way we engage in movement (physical activity) and non-movement behaviours (NMB; sleep and sedentary behaviours) (Fig. 1) [1]. For instance, the proportion of adults working in physically intense jobs reduced by 10 % between 1970 and 2000, computer use increased from 15 to 69 % between 1989 and 2009, and presently 29.2 % of Canadian adults watch 15 hours or more of television/week [2–4]. Overall, the majority of Canadian adults’ waking hours (~68 % of their day) are sedentary [5].

Morris’ study of the association of cardiovascular disease (CVD) with physical activity patterns set the stage for subsequent epidemiological studies on the subject [6]. In the 60 years since then, inquiry into the particulars of physical activity have led to an appreciation of it as a continuum [1], with types of physical activity identified on the basis of their intensity as measured in metabolic equivalents (METs) [7] or counts per minute (cpm) [5]. Accordingly, moderate (3–5.9 METs, 1535–3961 cpm) to vigorous (≥6 METs, ≥3962 cpm) levels of physical activity [5, 8] (MVPA) are most recommended based on their inverse associations with obesity and chronic disease risk [9–13], which has led to the current physical guidelines for adults: accumulation of at least 150 minutes of MVPA/week in bouts of 10 minutes or more [7, 14].

For the average Canadian, MVPA accounts for less than 5 % of the 24 hour day [5]. The rest of the day is comprised of other forms of physical activity and NMBs along the movement continuum (Fig. 1) [1]. These include light physical activity (1.6–2.9 METs, 100–1534 cpm; standing and walking slowly), sedentary behaviours (1–1.5 METs in a sitting or reclining posture, < 100 cpm), and sleep (0.9 METs) [1, 5, 8, 15, 16]. Sleep is an NMB that has reciprocal interactions with exercise [17] but, unlike sedentary behaviour, it also has an important role in physiologic function. Prospective studies have demonstrated the association of premature mortality with sitting time [18, 19], television and screen based activities [20–22], and other sedentary behaviours [23]. Similarly, short (≤5–6 h/day) and long (≥8–9 h/day) sleep duration was associated with greater odds of coronary heart disease (CHD) and stroke [24]. These two NMBs are distinct from light physical activity, which is highly negatively correlated with sedentary time, thereby suggesting a public health benefit of replacing sedentary time with light physical activity [25].

Metabolic Syndrome (MetS) has been suggested as an early indicator of chronic disease risk [26]. This condition describes the co-existence of several risk factors, which collectively contribute to an increased risk of CVD [27], diabetes [28, 29] and various cancers [30]. Physical activity levels have been associated with a higher risk of MetS overall, but also with its component risk markers [31]. The odds of MetS were 2.07, 95 % CI: 1.49–2.88 and 1.48, 95 % CI: 0.95–2.31 greater, in women and men respectively, among those watching ≥14 h/week, versus ≤ 7 h/week, of screen time [21]. Furthermore, short (≤6 h/day) sleep duration was associated with MetS (RR 1.74, 95 % CI: 1.05–2.72) [32]. Examining the independent associations of sleep time, screen time and sedentary time with MetS, Saleh et al. described a significant association for the highest level of the latter two behaviours [33]. When examining movement behaviours on the other end of the continuum, the inverse is noticed. Adults engaging in vigorous activity hours had half the odds (OR 0.52, 95 % CI: 0.40–0.67) of having MetS than among those who engaged in none, and moderate activity was similarly beneficial (OR 0.78, 95 % CI: 0.63–0.96 among those with ≥ 24 h of moderate METs/week, relative to <24 h) [34]. Even light physical activity was observed to associate with beneficial MetS outcomes (OR 0.44, 95 % CI: 0.24–0.81 in the highest and OR 0.51, 95 % CI: 0.29–0.89 in the middle tertile of light physical activity relative to the lowest) [35]. It is not surprising then that moderate and vigorous physical activity are suggested behaviours to manage MetS [36].

In light of the increasing prevalence of NMBs and their suggested risk for future chronic disease, this study examines the independent and joint associations of NMB with MetS. The stratified analysis of NMBs by MVPA guideline adherence presented here is a novel analysis that has not been described previously.

## Methods

### Data source

The Canadian Health Measures Survey (CHMS) is a nationally representative population survey created by Statistics Canada aimed at collecting information related to the health of Canadians [37]. Data collection involved a combination of general household interview and a visit to a mobile examination clinic (MEC). The former enabled collection of self-reported information regarding factors such as current health status, smoking habits, sleep, and alcohol use. The latter permitted collection of physical measures such as anthropometric data, accelerometer-based physical activity, and blood measures such as fasting plasma glucose levels.

Following their visit to a MEC, ambulatory respondents were asked to wear an Actical accelerometer (Phillips – Respironics, Oregon, USA) over their right hip on an elasticized belt during their waking hours for 7 days. At the first occurrence of midnight after the MEC visit, the accelerometers were initiated to monitor counts of physical activity [38]. The accelerometer measured and recorded time-stamped acceleration in all directions, which allows for calculation of the intensity, duration and frequency of movement [39]. The digitized values were summed over a user-specified interval or epoch of 1 min, resulting in a measure of cpm. Accelerometer based physical activity data were then cleaned to ensure individuals had 10 h of data per day, and at least 4 days of valid collection days. The monitors were returned to Statistics Canada, the data were downloaded and the monitor was checked to determine if it was still within the manufacturer's calibration specifications [5, 38].

### Study population

The full household sample study population from CHMS cycles 1 and 2 were pooled with data from the laboratory, fasted, and activity monitor subsamples (*N* = 9339). This combined study population was then restricted to only those individuals who participated in both the fasted and activity monitor subsample (*N* = 4273) [40]. Pregnant women (*N* = 10) and individuals under the age of 18 (*N* = 1362) were excluded, leading to the study population used for this analysis (*N* = 2901). Sample weights for the fasted subpopulation were applied to all analyses [40].

### Key definitions

#### Metabolic syndrome

The harmonized definition of MetS [41] was used to identify individuals with this condition on the basis of anthropometric measures, self-reported physician diagnosed risk conditions, or medication use related to the condition, and laboratory measures. More specifically, (1) ethnicity and gender specific waist circumference cut points, (2) elevated triglycerides, (3) reduced high-density lipoprotein cholesterol (HDL), (4) elevated blood pressure, and (5) elevated fasting plasma glucose were collectively used based on Harmonized definition specifications to identify MetS status. As per this definition, when an individual was identified to have 3 or more of the 5 risk markers, they were considered to have MetS.

#### Physical activity measures

Accelerometer based information allowed for the determination of daily minutes of moderate and vigorous physical activity per day [42]. MVPA guideline adherence was determined using the activity monitor based measure of daily minutes of moderate and vigorous physical activity and the current Canadian guidelines for adults. Full guideline adherence (≥150 mins of MVPA per week) and a threshold at half guideline adherence (75 min of MVPA per week) were used to create three levels of MVPA for analyses (less than ½ the guidelines as low MVPA, between ½ the guideline and guideline adherence as moderate MVPA, and more than or equal to guideline adherence as high MVPA) [14]. Sedentary time was also captured based on activity monitor based cpm [43], and levels were created by diving the data into thirds (<9.2 h/day, 9.2–10.3 h/day, ≥10.3 h/day). Sleep behaviours were identified based on self-reported hours of sleep over a 24 hour day, excluding time spent resting, and levels (based on thirds) were calculated as <7 h/day, 7–8 h/day, and ≥8 h/day. Screen time was measured using responses to questions measuring the typical number of hours per week that a respondent was in front of a computer, television, video game, or watching DVDs or videos over the last 3 months, which was then recoded to determine daily hours in these screen-based activities. From this, thirds were identified as <1.4 h/day, 1.4–2.1 h/day and ≥2.1 h/day. The reference categories were the lowest levels for sedentary time and sleep time, based on the better health outcomes at lower levels for these activities, and the 7–8 h/day level for sleep, based on its U-shaped association with ill health [44].

Combined variables were coded that took into account the levels of each NMB with levels of MVPA. In the case of sleep, for example, this led to a 9-category variable for sleep-MVPA (low sleep-low MVPA, low sleep-moderate MVPA, low sleep-high MVPA, moderate sleep-low MVPA, moderate sleep-moderate MVPA, moderate sleep-high MVPA, high sleep-low MVPA, high sleep-moderate MVPA, and high sleep-high MVPA). Similar variables were created for screen time and sedentary time.

#### Sociodemographic risk factors

Age, sex, and ethnicity (Caucasian vs. non-Caucasian) were sociodemographic risk factors used for this analysis based on self-reported responses. Sample size limitations did not permit more granularity on the basis of ethnic background. Income adequacy was derived by Statistics Canada based on self-reported total household income and number of individuals living within the household into a four category variable, which we then recoded into a three category measure for our analyses (lowest and lower middle income grouping, upper middle income grouping, and highest income grouping). Education level was based on self-reported highest attainment, and was coded as less than secondary education, secondary school graduate, and some post-graduate education or more.

#### Behavioural risk factors

Alcohol consumption was coded to align with definitions used internationally. This involved coding for the weekly number of grams of alcohol consumed based on self-reported number of drinks and conversion for a standard drink (1 drink = 14 g alcohol) [45, 46] so as to identify sex-specific categories of alcohol consumption: abstainer, average, hazardous, and harmful categories. Smoking patterns were coded using self-reported type of smoker and number of cigarettes per day among those who smoke. Non-smokers and former smokers were identified based on self-report, and current smokers were categorized as light, moderate and heavy smokers based on their daily number of cigarettes [47]. Time spent reading was included based on a self-reported measure, and daily minutes of light physical activity was included in analyses based on activity monitor based estimates.

### Analysis

Physical activity measures of interest were the NMBs of screen time, sedentary time and sleep time, as well as MVPA. Statistical analyses were conducted using SAS Enterprise Guide 6.1 (Cary, NC, US) and fasting sample weights for the combined CHMS cycles 1 and 2 were applied [48]. All analyses were weighted to account for unequal probabilities of selection and variance was calculated using the balanced repeated replicate weight approach. This method uses bootstrap weights to estimate sampling variability from complex stratified sampling. A sensitivity analysis was conducted to look at differences in the proportion of individuals adhering to MVPA guidelines who had an average of 11 h/day of accelerometer wear time, versus 16 h/day, and no significant differences were observed.

Levels for NMBs were created based on the 33^rd^ and 66^th^ percentile as determined through an analysis of the data distribution. Patterns along the various physical activity measures were described using mean estimates, and mean differences were determined using t-tests for independent samples. All other analyses were controlled for by confounders determined based on a p-value <0.5 for any of the four physical activity measures of interest. This led to the adjustment for age, alcohol consumption, and education level. Models were also adjusted for sex, income level, and smoking behaviours even though they were not significantly related to any of the four physical activity measures of interest.

The independent association of the four physical activity measures of interest with MetS were calculated using logistic regression analyses and included the remaining behaviours in the model. Effect modification, or moderation, was examined for the three NMBs by stratifying logistic regression results by the 3 levels of MVPA and controlling for the remaining NMBs [49, 50]. Combined associations were examined using the combined variables for each NMB with MVPA levels using logistic regression analyses with the referent category being the level with the least expected risk of MetS. These analyses also controlled for the covariates mentioned previously, including the remaining activity behaviours.

### Ethics approval

Approval was obtained from the University of Ottawa Research Ethics Board (File # H10-14-23).

## Results

When combining accelerometer and self-reported information to describe behaviours over the course of an average day, behaviours for 21 h of the day were captured, with waking hours accounting for 66 % of the day, and sleep for the remaining 34 %. On average, 46 % of Canadian adults’ hours were spent in sedentary time, and vigorous (0.2 %), moderate (1.5 %) and light (18.4 %) physical activity accounted for the remaining hours. Not including the 3 h that are unaccounted for based on data collection, Canadians spent 0.04 h/day in vigorous, 0.3 h/day in moderate, 3.9 h/day in light physical activity, and 9.7 h/day in sedentary time, as well as 7.2 h/day asleep.

**Fig. 2:**
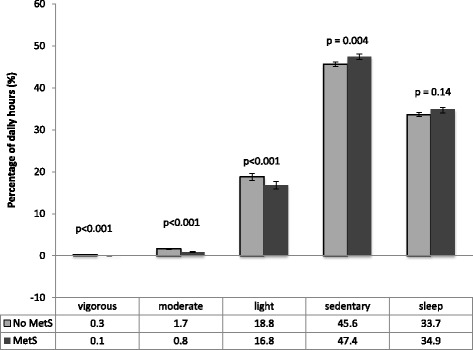
Percentage of daily hours spent in various forms of activity among those with or without MetS

MetS was prevalent in the Canadian adult population (19.0 %, 95 % CI: 15.7–22.4 %), and physical activity patterns varied by MetS status. Since the total hours of the day accounted for by MVPA and NMBs varied based on MetS status (21.23 hours for those without MetS and 20.97 hours for those with), results are discussed using percentage of total reported hours accounted for by the respective behaviours (Fig. 2). Comparing activity patterns between those with and without MetS, MetS was associated with significantly higher proportion of hours of sedentary time (*p* = 0.004), and significantly lower levels of vigorous (*p* < 0.001), moderate (*p* < 0.001) and light physical activity (*p* < 0.001). NMBs appear to be more prevalent among those with MetS, although sleep was not significantly different between those with and without MetS (sleep: *p* = 0.14).

These differences may be better understood through a comparison of the number of hours in different activities between both groups. Individuals without MetS spent 0.06 (95 % CI: 0.04-0.07) h/day in vigorous, 0.4 (95 % CI: 0.3–0.4) h/day in moderate, 4.0 (95 % CI: 3.8–4.2) h/day in light physical activity, and 9.7 (95 % CI: 9.6–9.8) h/day in sedentary time, as well as 7.2 (95 % CI: 7.0–7.3) h/day asleep, while individuals with MetS spent 0.01 (95 % CI: 0.00–0.02) h/day in vigorous, 0.2 (95 % CI: 0.2–0.2) h/day in moderate, 3.5 (95 % CI: 3.3–3.7) h/day in light physical activity, 9.9 (95 % CI: 9.8–10.1) h/day in sedentary time, as well as 7.3 (95 % CI: 7.1–7.5) h/day asleep.

Examining the independent relationships of physical activity behaviours with the odds of MetS, elevated odds was observed for those not achieving weekly MVPA guidelines (OR 2.9, 95 % CI: 1.9–4.5 for low MVPA, and OR 1.8, 95 % CI: 1.2–2.8 for moderate MVPA, as compared to those achieving MVPA guidelines). Similarly, excess screen time (≥2.1 h/ day, OR 1.8, 95 % CI: 1.2–2.8) was significantly associated with increased odds, as was excess sleep time (≥8 h/ day, OR 1.4, 95 % CI: 1.0–2.1) and moderate sedentary time (9.2–10.3 h/ day, OR 1.5, 95 % CI: 1.2–2.1) (Table 1).

**Table 1:** Independent relationships of MVPA and NMB on the risk of MetS

		*N*	Odds ratio
MVPA minutes	<75 mins/wk	1061	2.9 (1.9-4.5)
	75–150 mins/wk	707	1.8 (1.2–2.8)
	≥150 mins/wk	1133	ref
Screen Time	<1.4 h/d	1211	ref
	1.4–2.1 h/d	706	1.4 (0.9–2.1)
	≥2.1 h/d	982	1.8 (1.2–2.8)
Sleep Time	<7 h/d	957	1.2 (0.8–1.7)
	7–8 h/d	958	ref
	≥8 h/d	986	1.4 (1.0–2.1)
Sedentary Behaviour Time	<9.2 h/d	815	ref
	9.2–10.3 h/d	996	1.5 (1.2–2.1)
	≥10.3 h/d	1089	1.0 (0.7–1.4)

Models adjusted for age, sex, alcohol consumption, education level, income adequacy, and smoking behaviours. The respective remaining behaviours were included in the models so as to test independent relationships. (h/d = hours/day, mins/wk = minutes/week)

To examine whether the relationship between NMB and odds of MetS vary by adherence to MVPA guidelines, stratified analysis was employed and the remaining NMBs were kept in the model to test the independent effects. Indeed, the differential patterns of association for all NMBs across MVPA guideline strata suggest effect modification by the latter. Referent categories were created within each MVPA strata on the basis of the lowest risk category of NMB. Among individuals engaged in moderate MVPA, 1.4 to 2.1 and ≥ 2.1 h/day of screen time was significantly associated with an increased odds of MetS (OR 3.2, 95 % CI: 1.5–6.8 and 4.4, 95 % CI: 2.5–7.9 respectively). Similarly, among those engaged in low MVPA, there was a 1.7, 95 % CI: 1.1–2.5 increased odds of MetS for those who slept ≥8 h/day, and a 2.2, 95 % CI: 1.3–3.8 increased odds among those who engaged in 9.2–10.3 h of sedentary activity per day. There was no significant association of NMBs with MetS among those individuals who adhered to physical activity guidelines and accumulated 150 min of MVPA/week (Table 2).

**Table 2:** Effect modification of the risk of MetS associated with NMBs by MVPA guideline adherence

		Moderate to vigorous physical activity					
		<75 mins/wk		75–150 mins/wk		≥150 mins/wk	
		*N*	OR	*N*	OR	*N*	OR
Screen time	<1.4 h/d	383	ref	298	ref	530	ref
	1.4–2.1 h/d	256	1.3 (0.7–2.1)	179	3.2 (1.5–6.8)	271	1.0 (0.3–2.9)
	≥2.1 h/d	421	1.7 (0.9–3.3)	230	4.4 (2.5–7.9)	331	1.3 (0.6–2.6)
Sleep time	<7 h/d	315	1.2 (0.7–2.1)	202	1.1 (0.7–2.0)	298	1.3 (0.6–2.6)
	7–8 h/d	333	ref	247	ref	416	ref
	≥8 h/d	413	1.7 (1.1–2.5)	257	1.1 (0.5–2.4)	419	1.2 (0.5–2.8)
Sedentary behaviour time	<9.2 h/d	234	ref	215	ref	508	ref
	9.2–10.3 h/d	375	2.2 (1.3–3.8)	242	1.6 (0.9–3.0)	341	0.7 (0.3–1.6)
	≥10.3 h/d	452	1.6 (0.8–2.9)	250	0.5 (0.2–1.1)	284	1.0 (0.5–1.9)

Models adjusted for age, sex, alcohol consumption, education level, income adequacy, and smoking behaviours. The respective remaining behaviours were included in the models so as to test independent relationships. (h/d = hours/day, mins/wk = minutes/week)

**Fig. 3:**
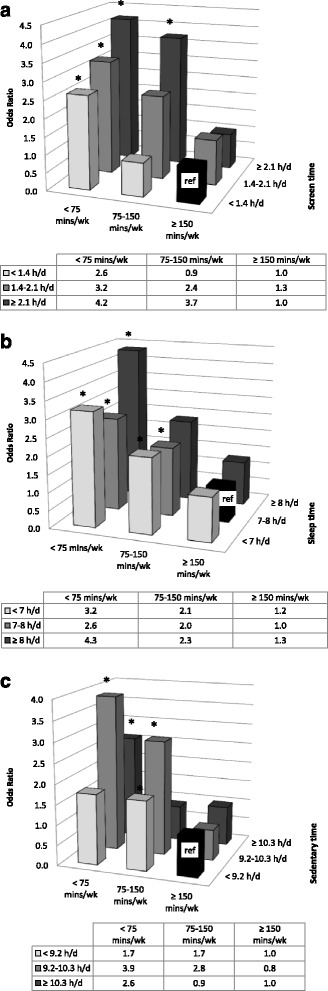
**a** Combined association of screen time and MVPA guideline adherence on MetS risk. Note: Models adjusted for age, sex, alcohol consumption, education level, income adequacy, smoking behaviours, sleep time, and sedentary time (h/d = hours/day, mins/wk = minutes/week, * = p<0.05). **b** Combined association of sleep time and MVPA guideline adherence on MetS risk. Note: Models adjusted for age, sex, alcohol consumption, education level, income adequacy, smoking behaviours, screen time, and sedentary time (h/d = hours/day, mins/wk = minutes/week, * = p<0.05). **c** Combined association of sedentary time and MVPA guideline adherence on MetS risk. Note: Models adjusted for age, sex, alcohol consumption, education level, income adequacy, smoking behaviours, sleep time, and screen time (h/d = hours/day, mins/wk = minutes/week, * = p<0.05)

To examine the joint association of NMB and MVPA behaviours, logistic regression analysis was conducted using variables reflecting all combinations of NMB and MVPA categories, as described in methods. The referent category was the one with lowest expected risk. For all NMBs, when MVPA guidelines were achieved, there were no increased odds of MetS regardless of level of the NMB (Fig. 3).

In the case of screen time, the referent category were those individuals who engaged in high MVPA and who accumulated <1.4 h of screen time per day. Relative to this group, all levels of screen behaviours for individuals who participated in low MVPA were associated with an increased odds of MetS (OR 2.6, 95 % CI: 1.4–4.8 for <1.4 h/day, OR 3.2, 95 % CI: 2.0–5.3 for 1.4–2.1 h/day, and OR 4.2, 95 % CI: 2.2–8.0 for ≥ 2.1 h/day). Among those who participated moderate MVPA, an increasing stepwise gradient was observed with increasing screen time (OR 0.9, 95 % CI: 0.5–1.8 for <1.4 h/day, OR 2.4, 95 % CI: 0.9–6.0 for 1.4–2.1 h/day, and OR 3.7, 95 % CI: 2.2–6.4 for ≥2.1 h/day). Finally, among those who adhered to MVPA guidelines, there were no significant associations of MetS with screen time (1.3, 95 % CI: 0.7–2.4 for 1.4–2.1 h/day, and 1.0, 95 % CI: 0.4–2.7 for ≥2.1 h/day) (Fig. 3a).

Sleep behaviour exhibited a U-shaped risk association with MetS, with the 7–8 h/day of sleep and MVPA guideline adherence used as the reference category. Similar to screen behaviours, all levels of sleep hours were associated with increased odds of MetS for individuals who achieved low MVPA (OR 3.2, 95 % CI: 1.6–6.5 for <7 h/day, OR 2.6, 95 % CI: 1.4–4.9 for 7–8 h/day, and OR 4.3, 95 % CI: 2.1–9.1 for ≥ 8 h/day). Among those who achieved moderate MVPA, associations were only significant for those with moderate or low sleep time (OR 2.1, 95 % CI: 1.1–4.1 for <7 h/day, OR 2.0, 95 % CI: 1.0–3.8 for 7–8 h/day, and OR 2.3, 95 % CI: 0.9–6.2 for ≥ 8 h/day). There were no significant associations of MetS with sleep time among those who adhered to MVPA guidelines, (1.2, 95 % CI: 0.6–2.4 for <7 h/day, and 1.3, 95 % CI: 0.6–2.6 for ≥ 8 h/day) (Fig. 3b).

Finally, when examining sedentary time, there appears to be an inverse U-shaped curve, with individuals engaged in 9.2–10.3 h/day of sedentary time at most risk of MetS compared to the referent category of <9.2 h/day of sedentary time and MVPA guideline adherence. Among those who achieved low MVPA, sedentary time in excess of 9.2 h each day were associated with MetS odds (OR 1.7, 95 % CI: 0.9–3.4 for <9.2 h/day, OR 3.9, 95 % CI: 2.1–7.1 for 9.2–10.3 h/day, and OR 2.6, 95 % CI: 1.5–4.6 for ≥ 10.3 h/day). Achieving moderate MVPA was only associated with increased odds of MetS for those in the moderate category of sedentary time (OR 1.7, 95 % CI: 1.0–3.0 for <9.2 h/day, OR 2.8, 95 % CI: 1.5–5.4 for 9.2–10.3 h/day, and OR 0.9, 95 % CI: 0.4–1.7 for ≥ 10.3 h/day). For those who did achieve MVPA guidelines, the associations of sedentary time with MetS were not significant (0.8, 95 % CI: 0.4–1.6 for 9.2–10.3 h/day and 1.0, 95 % CI: 0.6–1.7 for ≥ 10.3 h/day) (Fig. 3c).

## Discussion

This article explores the associations of MVPA and NMBs with MetS. The findings suggest that adhering to MVPA guidelines may mitigate MetS risk. MetS was prevalent in the Canadian adult population [51], with almost one in five meeting criteria. This finding parallels other Canadian studies using the Harmonized definition for MetS [51, 52], but is higher than the 14.9 % prevalence reported using the NCEP definition [51, 53], likely due to the fact that the former uses sex- and ethnicity- specific cut-offs.

Canadian adults spend approximately two-thirds of their day awake, and of these hours, a majority of time is spent in sedentary activity. Although roughly 9.7 h/day, or 66 % of waking hours in sedentary time, reflects previous Canadian reports [5], it is noteworthy that such behaviour comprises a greater proportion of waking hours than those reported for Australia (57 %) [25] or the U.S. (56.8 % [54] to 58 % [3]). This is concerning given the suggestion that time spent in sedentary behaviours displaces time spent in lower intensity movement behaviours, such as light physical activity, and can result in an overall reduction in physical activity levels [55]. Not surprisingly, individuals with MetS were more likely to engage in sedentary behaviours than those without MetS, and the reciprocal was true for active behaviours. In an examination of physical activity patterns with a clustered metabolic risk score, Healy et al. also described a similar differential pattern of physical activity based on MetS status(objectively assessed sedentary behaviour β-estimate 0.23, 95 % CI: 0.08 to 0.38, light physical activity β-estimate −0.20, 95 % CI: −0.35 to −0.04, MVPA β-estimate −0.17, 95 % CI: −0.34 to −0.01) [25].

Given that movement and NMB do indeed associate with MetS, the independent and combined associations of these activity levels were of interest. Independent of remaining NMB behaviours, this study found that MVPA and all NMBs examined are significantly associated with an increased odds of MetS. Similar to this study, Clarke et al. found that weekly volume of MVPA was strongly associated with MetS. Their findings, like ours, suggest the benefits of adhering to weekly MVPA guidelines. While our suggestion is based on testing independent associations of weekly MVPA, their study also examined the daily patterning of MVPA and did not find an association of frequency of MVPA with MetS, but did find one with weekly volume of MVPA [56]. Similarly, the independent association of MVPA with MetS has been described elsewhere [57]. With regard to screen time, its association with MetS independent of physical activity has also been described previously for adolescents [58] and adults [57].

It is noteworthy that sedentary time was a significant predictor of MetS, given previous cross-sectional [25] and prospective [59] reports suggesting it would be. The lack of graded association, however, may be due to the absence of considering the ways in which this time was accumulated. That is to say, it may be that not total sedentary time, but rather bouts and length of bouts of sedentary time, are what is important for preventing MetS. In their assessment of this phenomenon, Healy et al. reported that the majority of sedentary activity breaks fell under light physical activity behaviours, and that independent of total sedentary time, the intensity and frequency of these breaks were negatively associated with MetS [60]. While the absence of bout data limited considerations of objective sedentary time, it is worth noting that screen time behaviours did associate with MetS as anticipated and that these behaviours often represent prolonged sedentary time. The independent association of sleep is also worth considering. Sleep levels identified in this analysis reflected recommended sleep durations, and portrayed a U-shaped pattern [61]. The finding that excess sleep, and not less sleep, was significantly associated with MetS may be due to the fact that we considered independent associations, which suggests that previous reported associations of sleep with MetS were possibly affected by other physical activity patterns [44].

Given reports that sedentary behaviours appear to be increasing among adults [62], we sought to explore whether MVPA behaviour moderates the association of NMBs with MetS. Through stratified analysis examining the associations of MVPA levels on their association, significant associations were observed for NMBs at levels below MVPA guideline achievement. Most noteworthy is how, for any level of NMB, if adherence to physical activity guidelines was achieved, then no association of NMB with MetS was observed. The differential patterns, whether significant or not, do suggest a moderating role of guideline adherence. However, given that the independent associations of NMBs on MetS were non-significant in some instances, it is not surprising that the moderating associations by MVPA on these behaviours were not significant either.

While the stratified analysis enabled within strata comparisons, combined analyses permitted comparisons of 8 levels of NMB-MVPA with the NMB-MVPA level associated with the least odds of MetS. Patterns observed within strata in Table 2 persisted in Fig. 3. However, Fig. 3 permitted across strata comparisons, which appear to be more informative. Indeed, among participants adhering to MVPA guidelines, no elevated odds for MetS for any NMB was observed. While sedentary time also showed significant associations, the unexpected inverse U-shaped pattern suggests that more study is required to better understand this pattern of results. As mentioned previously, the lack of consideration made for breaks in sedentary time may obscure true representation of sedentary behaviour. Nevertheless, in a prospective analysis of the combined association of MVPA and leisure-time sitting, which represents a sedentary behaviour, Bell et al. reported 5- and 10- year estimates of incident MetS that mimic the inverse U-shape we report here. They suggest this inverse U-shape may be attributed to misclassification error due to changing behaviour patterns over time [63]. In contrast, a similar prospective analysis reported a linear trend risk of MetS with sedentary behaviour [59]. Therefore, further examination into the associations of sedentary behaviour, MVPA and MetS may be warranted.

Continuing the examination of combined associations, screen time and sleep time yielded some significant associations. Examining their joint associations in relation to obesity, Dunton et al. described the interaction associations between MVPA and TV or movie time [64]. For both screen time and sleep time, achieving less than half the guideline per week was associated with an increased risk. These results give credence to the importance of achieving MVPA guidelines regardless of NMB behaviours, much as was observed in the present analysis.

Physical activity has been supported for the primary and secondary prevention of a number of chronic conditions [65–68], including MetS [36]. Based on a review of the literature, no previous study of MetS was identified to have examined the utility of MVPA guideline adherence independent of NMB, or to have examined its combined utility across different NMB behaviours in adults. As a result, the present study is a novel analysis presenting the importance of MVPA guideline adherence in the context of NMB behaviours and MetS risk. As with a prior study examining the combined associations of sedentary behaviour with MVPA in children with regard to MetS [69], this study highlights the potential importance of MVPA guideline adherence independent of NMB behaviours in adults.

### Limitations

This study used pooled cycles of the cross-sectional CHMS survey and it is possible that patterns of physical activity and NMBs changed over the four-year time frame. The cross-sectional nature of the study also restricted exploration of temporality of events. Furthermore, much of the literature on MetS uses the NCEP definition for MetS, while our study used the Harmonised definition. Differences in these definitions on the basis of ethnic and sex-specific cut-offs may account for differences between studies.

The combination of measured and self-report information might account for the missing 3 hours from the full 21 hour day used in this analysis. This is possibly due to reporting bias for self-reported sleep data. However, it is possible that accelerometer data may also provide for some error. The accelerometer used in this study was meant to be worn during waking hours, and valid data were identified based on having at least 4 days of valid days, i.e., days with a minimum of 10 h of wear time. This guideline is widely used, but was originally not developed using empirical evidence [39]. Failing to wear the accelerometer during the full waking period may have thus affected results. This is important since accelerometers worn for too few hours may lead to underestimation of time spent in different intensities of physical activity. In an semi-simulated analysis of the adequacy of wear times for different physical activity intensities, it was shown that wear times at 10 h/day resulted in 30 % less recorded time spent in inactivity relative to the reference period of 14 h/day [39]. Accelerometer data capture for sedentary and light physical activity are also affected by the epoch duration of data capture, which might misclassify record of physical activity in either category as a result, resulting in very different interpretations. This too may have affected sedentary estimates in this study [38, 70]. To address this concern, a sensitivity analysis was conducted, and did not suggest variation based on wear-time. The inability to access information regarding sedentary breaks may too have limited the accurate representation of sedentary risk for MetS [60, 71]. Future studies should look further into optimizing data collection for sedentary time, including information regarding bouts of sedentary time and addressing possible misclassification concerns.

## Conclusions

Adhering to physical activity guidelines may mitigate the associations of NMBs with MetS. Given that associations between NMBs and MetS were not significant among Canadians meeting PA guidelines, these findings suggest the beneficial role of physical activity to prevent chronic disease risk.

## Abbreviations

cpm: counts per minute
CVD: cardiovascular disease
MEC: mobile examination clinic
METs: metabolic equivalents
MetS: metabolic syndrome
MVPA: moderate to vigorous physical activity
NMB: non-movement behaviour
PA: physical activity
